# Supplementary material for: Illustration of the Importance of Adjustment for within- and between-Person Variability in Dietary Intake Surveys for Assessment of Population Risk of Micronutrient Deficiency/Excess Using an Example Data Set
Source: Nutrients. 2022 Jan 11;14(2):285. doi: 10.3390/nu14020285 (PMC8781123; doi:10.3390/nu14020285)
Supplement: Supplementary file 1 [file nutrients-14-00285-s001.zip › nutrients-1516337-supplementary.pdf]

Supplementary Tables S1–S3 and Supplementary Figures S1–S2: Results for energy and macronutrients

Mean usual energy, carbohydrate, protein and fat intake were similar in all three age groups when compared to Day-1 values. Median usual intakes of carbohydrate, protein and fat intakes were significantly higher in the 3–<6-year-olds and 6–<10-year-olds, but only median fat intake was significantly higher in the 1–<10-year-olds when compared to Day-1 values. Median energy intake was significantly higher in the 6–<10-year-olds. It is evident from Table S.1 that the percentage difference between median and usual Day-1 intakes is less than 5% for most of the median comparisons. For the median carbohydrate intake in 6–<10-year-olds and median fat intake in 1–<3-year-olds and 3–<6-year-olds the percentage difference is between 5% and 9%.

Table S1 Mean (SE of the mean), median (SE of the median) and percentage difference (usual intake versus Day-1 intake) for energy, carbohydrate, protein, and fat intake by age group

		Age 1–<3 years n=333			Age 3–<6 years n=514			Age 6–<10 years n=479		
**Nutrient**		Usual intake	Day-1 intake	% Diff	Usual intake	Day-1 intake	% Diff	Usual intake	Day-1 intake	% Diff
Energy (kJ) (λ=0.13)	Mean (SE of mean)	4933 (190)	4950 (145)	-0.3	5623 (146)	5624 (158)	-0.02	6528 (85)	6547 (138)	-0.3
	Median (SE of median)	4795 (147)	4727 (224)	1.4	5459 (132)	5293 (137)	3.1	6341 (124)	6266.6 (119)	1.2 *
Carbohydrate (g) (λ=0.10)	Mean (SE of mean)	163.7 (6.1)	164.3 (5.3)	-0.4	187.3 (2.4)	187.0 (6.1)	0.2	209.0 (1.6)	209.7 (4.4)	-0.3
	Median (SE of median)	156.7 (6.3)	150.9 (5.0)	3.8	182.4 (2.4)	171.3 (6.9)	6.5 ***	202.0 (3.3)	199.8 (6.1)	1.1 *
Protein (g) (λ=0.39)	Mean (SE of mean)	34.7 (1.5)	34.8 (1.1)	-0.3	40.4 (1.2)	40.5 (1.1)	-0.3	46.3 (1.3)	46.5 (1.1)	-0.4
	Median (SE of median)	33.9 (1.6)	33.0 (1.6)	2.7	39.8 (1.2)	38.6 (1.3)	3.1 **	45.9 (1.4)	43.9 (1.3)	4.6 *
Total fat intake (g) (λ=0.30)	Mean (SE of mean)	39.4 (1.9)	39.4 (1.6)	0.0	44.0 (2.1)	44.2 (1.8)	-0.5	56.3 (1.3)	56.4 (1.9)	-0.2
	Median (SE of median)	38.2 (1.6)	35.5 (2.0)	7.6 **	41.4 (1.8)	38.3 (1.8)	8.1 **	54.4 (1.9)	52.5 (1.7)	3.6 **

Day-1 intake: The reported 24-hour recall on the first visit.
Usual intake: long-term daily average intake as calculated using the NCI amount-only method.
There were no significant differences between the means of the Day-1 intake and the usual intake, *t*-test, *p*-values>0.05.
*p<0.05; **p<0.01; ***p<0.001: Significant difference between the locations of Day-1 and usual intakes, Kruskal-Wallis test.

Table S2 shows that a larger mean bias (λ<0.15) is present for energy and carbohydrate, but that the ratio of within-person to between-person variance is well below 10 for energy and macronutrient intakes in all three age groups.

Table S2: Within-person and between-person variance, ratio of these two parameters and the coefficient of variation for usual intake of energy, carbohydrate, protein and fat intake, by age group

Nutrient (Box-Cox TP)	Age: 1–<3 years				Age: 3–<6 years				Age: 6–<10 years			
	Var_e *	Var_u	Ratio	CV(%)	Var_e	Var_u	Ratio	CV(%)	Var_e	Var_u	Ratio	CV(%)
Energy (kJ) (λ=0.13)	0.73	0.59	1.24	3.8	0.81	0.62	1.31	2.6	0.61	0.64	0.95	1.3
Total carbohydrates (g) (λ=0.13)	0.25	0.33	0.76	3.7	0.44	0.22	2.00	1.3	0.27	0.27	1.00	0.8
Total fat (g) (λ=0.28)	2.09	0.46	4.54	4.8	1.57	1.29	1.22	4.8	1.80	0.92	1.96	2.3
Total protein (g) (λ=0.43)	3.09	1.20	2.58	4.4	3.19	0.93	3.43	2.9	3.44	0.74	4.65	2.8

A λ-value <0.15 reflects a larger mean bias because of sensitivity to the transformation applied [16]. Lambda values associated with the first execution or base run of the macros are reported and are the same across the three age groups.
*Var_e: Within-person variance; Var_u: Between-person variance; Ratio=Var_e/Var_u, the ratio of within-person to between-person variance.
CV: Coefficient of variation of the mean of usual intake calculated as follows:
CV=100 × (standard error of the mean)/mean.
TP: Transformation parameter

Comparison of the percentage children with an energy intake below the EER shows that Day-1 intake results overestimated deficiency risk (%(Day-1 intake < EER) minus %(usual intake < EER)) in 1–<3-year-olds with 10.5%, underestimates risk in the 3–<6-year-olds with 3.8% with the difference being less than 1% in in 6–<10-year-olds (Table 4; Figure 1). For %E intake from carbohydrate overestimation of intake less than the lower limit of the AMDR range by Day-1 intake when compared to usual intake was 11.1% in 1–<3-year-olds, 6.8% in 3–<6-year-olds and 8.3% in 6–<10-year-olds. For %E from protein, Day-1 intake results overestimated intake less than the lower limit of the AMDR range with 2% in 1–<3-year-olds, 15.6% in 3–<6-year-olds and 27.2% in 6–<10-year-olds range, but in the youngest two age groups the difference was less than 4%, while it was 21.7% in the oldest age group.

For %E from carbohydrate, Day-1 intake results overestimated intake above the upper limit of the AMDR range with 19.4% in 1–<3-year-olds, 11.1% in 3–<6-year-olds and 15.6% in 6–<10-year-olds. For %E from protein, Day-1 intake results overestimated intake above the upper limit of the AMDR range with less than 3% in the younger two age groups and 9.8% in 6–<10-year-olds. For %E from fat, Day-1 intake results overestimated intake above the upper limit of the AMDR range with less than 10% in the two younger age groups (9.8% and 9.7% respectively), while it was 12.4% in 6–<10-year-olds.

Table S3: Comparison of percentage below EER for energy, as well as percentage below the lower limit of and percentage above the upper limit of the AMDR range, by age group

		Day-1 intake		Usual intake		Difference[1] %<EER or lower limit of AMDR (%>upper limit of AMDR)
	Age group	%< EER or lower limit of AMDR range (95% CI)	%>upper limit of AMDR range (95% CI)	%< EER or lower limit of AMDR range (95% CI)	%>upper limit of AMDR range (95% CI)	
Energy (kJ) EER (Male / female): 1-2 years=4393 / 4166; 3 years=6213 / 5837; 4 years=6552 / 6171; 5 years=6937 / 6514; 6 years=7289 / 6870; 7 years= 7699 / 7192; 8 years=8079 / 7573; 9 years=8548 / 7908	1–<3 years (n=333)	43.3 (35.0-51.6)	-	32.8 (28.3-37.3)	-	-10.5% (-)
	3–<6 years (n=514)	67.4 (60.5-74.2)	-	71.2 (61.1-81.2)	-	3.8% (-)
	6–<10 years (n=479)	75.1 (70.3-79.9)	-	74.4 (68.8-79.9)	-	-0.7% (-)
%E from Carbohydrates AMDR-interval (1–<10 years): 45%–65%	1–<3 years (n=333)	11.5 (7.4-15.6	30.7 (25.2-36.2)	0.4 (0.0-4.0)	11.3 (6.9-15.7)	-11.1% (-19.4%)
	3–<6 years (n=514)	7.3 (4.4-10.2)	33.0 (27.4-38.7)	0.5 (0.0-2.9)	21.9 (8.5-35.3)	-6.8% (-11.1%)
	6–<10 years (n=479)	8.8 (5.8-11.9)	22.8 (18.0-27.6)	0.5 (0.0-2.2)	7.2 (0.0-19.2)	-8.3% (-15.6%)
%E from Protein AMDR interval: 1–3years=5%–20%; 4–9years=10%–30%	1–<3 years (n=333)	2.0 (0.0-4.1)	2.8 (0.3-5.3)	0.0 (-)	0.01 (0.0-0.2)	-2.0% (-2.79%)
	3–<6 years (n=514)	18.3 (14.8-21.8)	2.5 (1.0-4.0)	2.7 (0.0-6.1)	0.0 (-)	-15.6% (-2.5%)
	6–<10 years (n=479)	30.5 (24.4-36.7)	0.03 (0.0-0.1)	3.3 (0.0-23.7)	0.0 (-)	-27.2% (-0.03)
%E from Fat AMDR interval: 1–3years=30%–40%; 4–9years=25%–35%	1–<3 years (n=333)	52.4 (45.6-59.3)	14.2 (9.5-18.8)	50.7 (40.4-60.9)	4.4 (0.0-10.8)	-1.7% (-9.8%)
	3–<6 years (n=514)	43.3 (37.0-49.6)	18.5 (13.6-23.3)	40.2 (30.5-49.9)	10.8 (7.5-14.0)	-3.1% (-7.7%)
	6–<10 years (n=479)	26.3 (20.5-32.0)	38.1 (32.1-44.1)	4.6 (0.0-17.6)	25.7 (13.4-38.1)	-21.7% (-12.4%)

AMDR=Acceptable macronutrient range; EER=Energy-efficiency ratio; SE=standard error.
Day-1 intake: The reported 24-hour recall on the first visit. Calculations were made using sample weights and the complex survey design.
Usual intake: long-term daily average intake as calculated using the NCI amount-only method, using BRR weights.
[1]Difference: %(usual intake < EER) minus %(Day-1 intake < EER) for energy; %(usual intake <lower limit of the AMDR range) minus % (Day-1 intake %< lower limit of the AMDR range) for carbohydrate, protein and fat; and %(usual intake > upper limit of the AMDR range) minus % (Day-1 intake %> upper limit of the AMDR range) for carbohydrate, protein and fat.

Figure S1 presents the density functions of Day-1 and usual intakes of the macronutrients within the context of DRI cut-points to further illustrate the results presented in Table S1, Table S2 and Table S3, with the difference in risk of deficient energy and macronutrient intake outside the AMDR-range for %E from carbohydrate, protein and fat clearly illustrated in Figure S2.

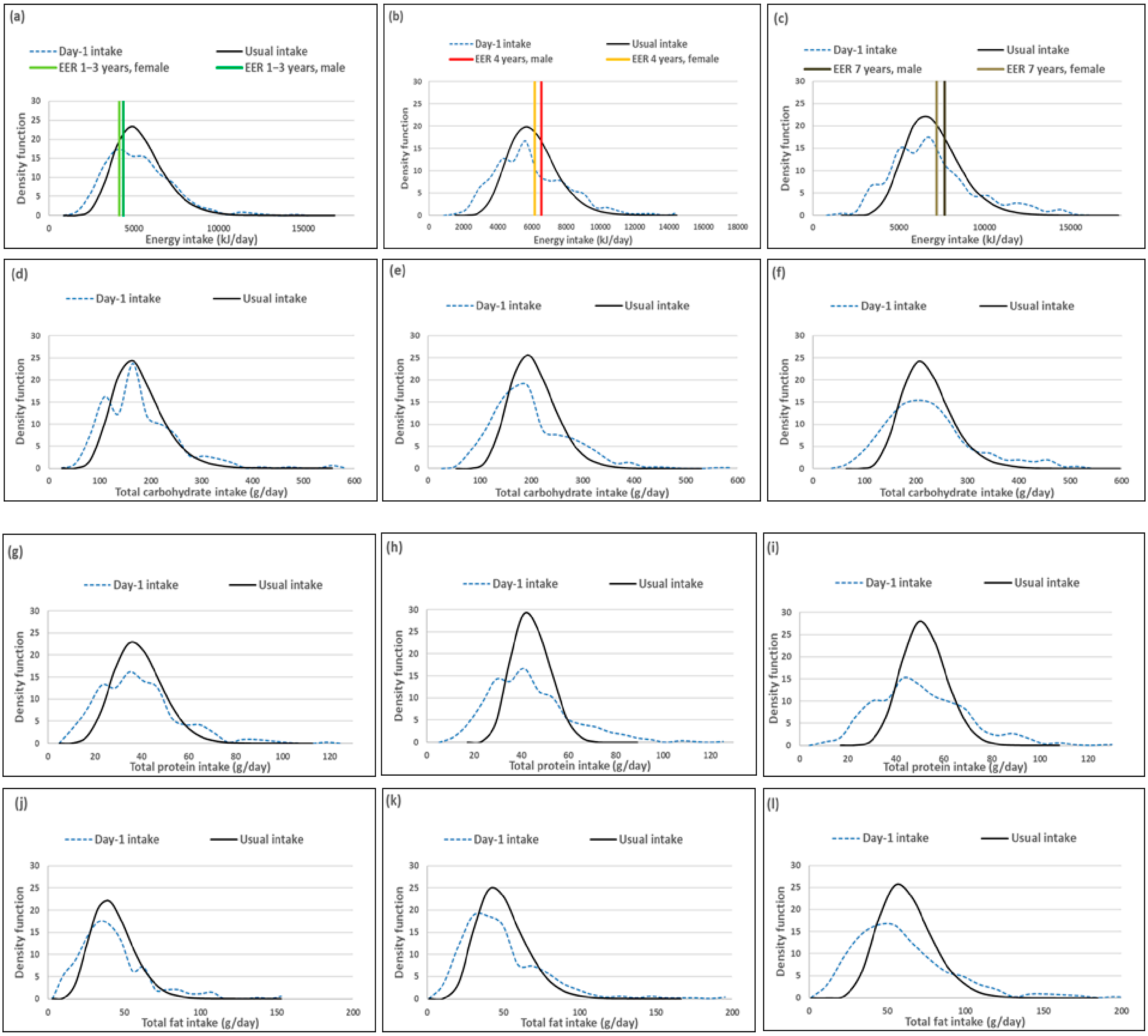

Figure S1: Distributions of the simulated, back-transformed values (usual intake) and the distribution of Day-1 intakes of energy, carbohydrate, total protein and total fat, by age group. (a): Age 1–<3 years: Energy intake; (b): Age 3–<6 years: Energy intake; (c): Age 6–<10 years: Energy intake; (d): Age 1–<3 years: Carbohydrate intake; (e): Age 3–<6 years: Carbohydrate intake; (f): Age 6–<10 years: Carbohydrate intake; (g): Age 1–<3 years: Total protein intake; (h): Age 3–<6 years: Total protein intake; (i): Age 6–<10 years: Total protein intake; (j): Age 1–<3 years: Total fat intake; (k): Age 3–<6 years: Total fat intake; (l): Age 6–<10 years: Total fat intake.

Figure S2 demonstrates how the spread of the usual intake density function of %E from carbohydrate, protein and fat becomes narrower when the within-person variance is addressed. The narrowing of the spread of the usual intake density function is evident for all three macronutrients, for all three age groups, but is specifically prominent in the 6–<10-year-olds.

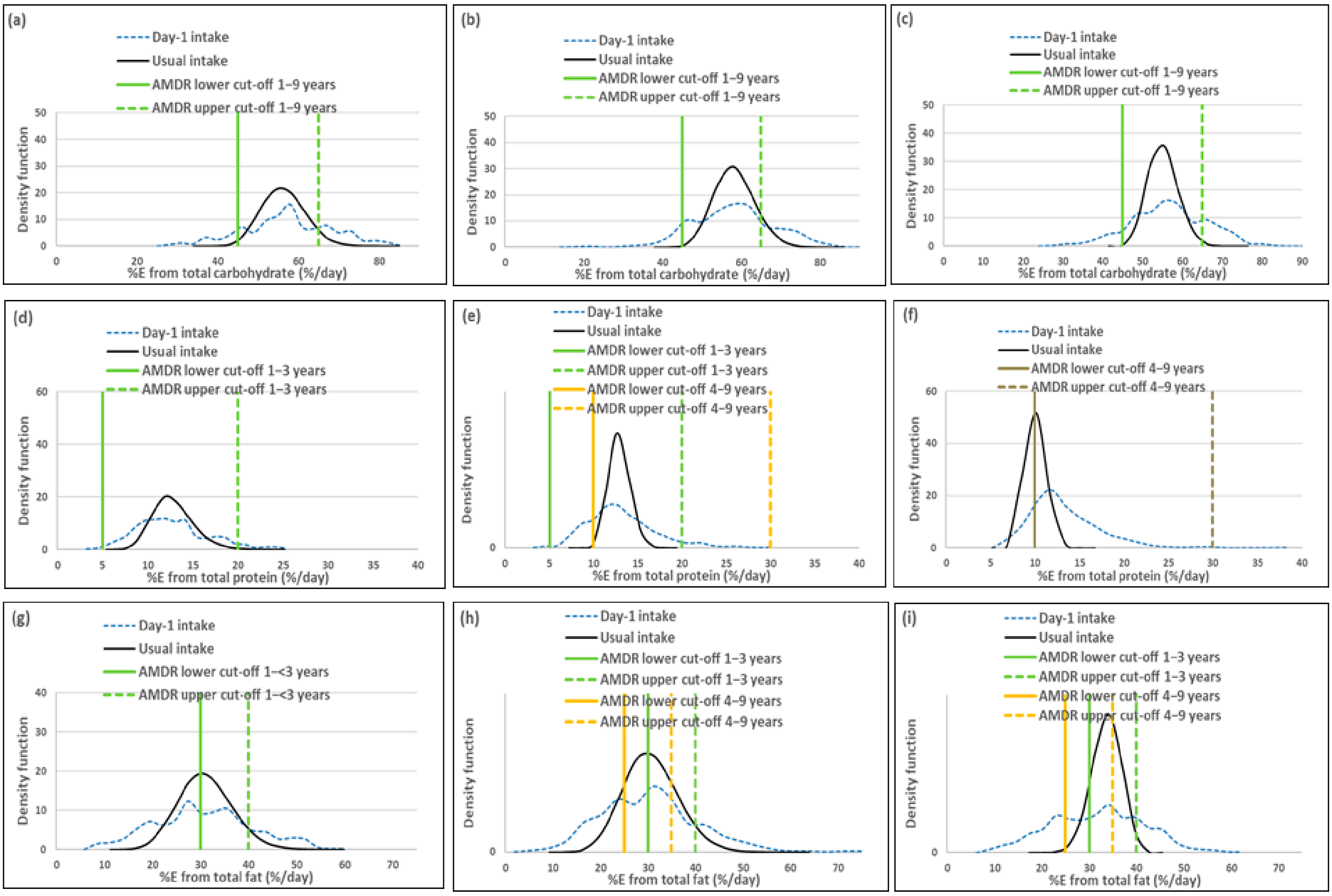

Figure S2: Distributions of the simulated, back-transformed values (usual intake) and the distribution of Day-1 intakes within the context of AMDR-values of %E from carbohydrate, %E from total protein and %E from total fat, by age group. (a): Age 1–<3 years: %Energy from total carbohydrate intake; (b): Age 3–<6 years: %Energy from total cabohydrate intake; (c): Age 6–<10 years: %Energy from total carbohydrate intake; (d): Age 1–<3 years: %Energy from total protein intake; (e): Age 3–<6 years: %Energy from total protein intake; (f): Age 6–<10 years: %Energy from total protein intake; (g): Age 1–<3 years: %Energy from total fat intake; (h): Age 3–<6 years: %Energy from total fat intake intake; (i): Age 6–<10 years: %Energy from total fat intake.
